# Exploring the role of cathepsins in sarcopenia-related traits: Insights from a Mendelian randomization study

**DOI:** 10.1097/MD.0000000000042700

**Published:** 2025-06-06

**Authors:** Yiwen Hou, Huaxin Deng, Wei Liu, Yan Liu

**Affiliations:** a School of Public Health, Hengyang Medical School, University of South China, Hengyang, China; b Department of Occupational Health and Radiation Health, Chongqing Center for Disease Control and Prevention, Chongqing, China; c Department of Public Health, Tongji Hospital, Tongji Medical College, Huazhong University of Science and Technology, Wuhan, China.

**Keywords:** appendicular lean mass, cathepsins, hand grip strength, Mendelian randomization, sarcopenia

## Abstract

Sarcopenia, a degenerative loss of skeletal muscle mass, strength, and function, poses a significant public health issue, particularly among aging populations. Cathepsins have recently emerged as key regulators of muscle metabolism and potential contributors to sarcopenia. This study employed Mendelian randomization (MR) to elucidate the causal relationships between 10 specific cathepsins and sarcopenia-related phenotypes, particularly appendicular lean mass (ALM) and handgrip strength, to identify potential therapeutic targets for sarcopenia intervention. A two-sample MR approach was used, employing genetic variants as instrumental variables to explore the impact of cathepsins on ALM and handgrip strength. ALM-related genetic association data were sourced from the UK Biobank, whereas grip strength data were derived from a meta-analysis focused on muscle weakness. Cathepsin levels were obtained using data from the INTERVAL study. The primary analysis method was the inverse variance weighted method, supplemented by MR-Egger, simple mode, weighted median, and weighted mode analyses to confirm the robustness of the findings. Positive associations of cathepsin S and E with ALM were demonstrated, whereas negative associations were observed for cathepsin F and B. No significant associations were identified between any cathepsin and grip strength in the primary inverse variance weighted analysis; however, secondary analyses indicated that cathepsin S may serve as a potential risk factor for reduced grip strength. Cathepsins S, E, F, and B were identified as playing significant roles in regulating muscle mass and strength, with cathepsin S potentially affecting grip strength. Targeting these enzymes may offer a viable strategy for developing treatments for sarcopenia. Although causal insights were provided, limitations include reliance on genetic data predominantly from European populations and the use of summary-level data, which constrains generalizability and hinders exploration of individual-level variability and molecular mechanisms. Future studies should validate these findings across diverse populations and investigate the effects of cathepsin on muscle health.

## 1. Introduction

Sarcopenia, a progressive and generalized skeletal muscle disorder, is characterized by loss of muscle mass, strength, and function.^[1]^ Affecting 10% to 16% of older adults worldwide, sarcopenia represents a major public health concern, significantly increasing risks of falls, fractures, physical disability, and mortality, and is associated with a broad spectrum of adverse health outcomes.^[2,3]^ Sarcopenia-related traits, such as appendicular lean mass (ALM) and handgrip strength, serve as critical indicators for diagnosing and assessing sarcopenia severity.^[4]^ Low ALM and reduced grip strength are linked to poor physical performance and increased frailty, underscoring the importance of identifying factors influencing these traits to develop effective interventions.^[5]^

Cathepsins, a family of proteolytic enzymes belonging to the lysosomal cysteine protease group, are essential for protein degradation, antigen presentation, and apoptosis.^[6,7]^ These enzymes are implicated in various physiological and pathological processes, including cancer, autoimmune diseases, cardiovascular diseases, and neurodegenerative disorders.^[8,9]^ Recent evidence suggests that cathepsins play critical roles in muscle metabolism and function, with direct relevance to sarcopenia. Altered cathepsin activity has been associated with muscle wasting and inflammation, both key components of sarcopenia.^[10]^ For instance, increased expression and activity of calpain and cathepsin B, L, and H in muscle tissues of patients with polymyositis or dermatomyositis suggest that these proteases play a pivotal role in the degradation of muscle fibers in inflammatory myopathies.^[11]^

The potential association between cathepsins and sarcopenia-related traits represents an emerging research area. Cathepsins may influence muscle mass and strength through their roles in protein turnover, inflammation, and cellular signaling pathways.^[12,13]^ Establishing causal relationships between specific cathepsins and sarcopenia-related traits could provide novel insights into the mechanisms underlying muscle degeneration and help identify new therapeutic targets. However, observational studies are often limited by confounding factors and reverse causation, which can obscure true causal effects.

Mendelian randomization (MR), an analytical approach using genetic variants as instrumental variables, enables inference of causal relationships between exposure and outcomes.^[14,15]^ Using single-nucleotide polymorphisms (SNPs) associated with cathepsin levels as instrumental variables, MR can help overcome the limitations of observational studies, providing more reliable evidence of causality.^[16,17]^ This method leverages the random assignment of genetic variants during gamete formation, resembling the randomization process in controlled trials.^[18]^ A two-sample MR design was employed to investigate the causal effects of 10 cathepsins on sarcopenia-related traits, specifically ALM and handgrip strength, allowing assessment of cathepsins’ roles in sarcopenia with enhanced confidence and precision.

The primary aim of this study was to examine the causal relationship between cathepsins and sarcopenia-related traits through MR analysis. Identification of specific cathepsins influencing muscle mass and strength may elucidate novel mechanisms and potential therapeutic targets for sarcopenia. Genome-wide association study (GWAS) summary data, selection of genetic instrumental variables, and various MR statistical analyses were utilized to ensure robust and reliable findings. Insights gained may pave the way for new strategies to combat sarcopenia and improve the quality of life for affected individuals.

## 2. Materials and methods

### 2.1. Study design

A two-sample MR analysis was conducted to investigate the potential causal effects of 10 cathepsins on sarcopenia. SNPs were utilized as instrumental variables to represent levels of the 10 cathepsins. By employing SNPs as instrumental variables, the impact of potential confounding factors was minimized, thereby enhancing the reliability of causal inference results.

**Figure 1.**
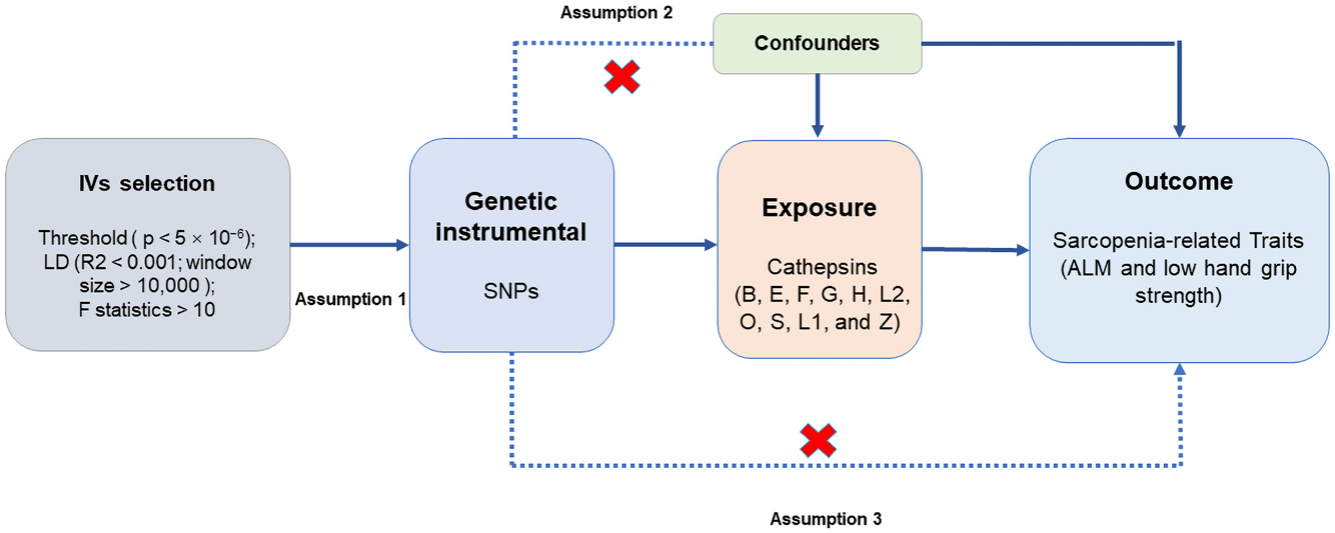
Flow chart of the study design and the assumption of Mendelian randomization analysis to investigate the potential causal effects of 10 cathepsins on sarcopenia.

The MR analysis was based on 3 key assumptions (Fig. 1). First, the selected instrumental variables (SNPs) were strongly associated with cathepsin levels. Second, the instrumental variables were independent of confounding factors that could bias the estimation of causal effects. Third, the instrumental variables influenced outcomes solely through their impact on the exposure of interest.

### 2.2. Data sources

Sarcopenia, as defined by the European Working Group on Sarcopenia in Older People (EWGSOP), is characterized by loss of muscle mass, strength, and function.^[19]^ ALM and handgrip strength serve as measures of muscle mass and strength, respectively, in older adults. Accordingly, ALM and low handgrip strength were selected as sarcopenia-related traits for MR analysis.

ALM-related GWAS summary statistics were obtained from the UK Biobank GWAS database, encompassing 450,243 participants. The UK Biobank, one of the world’s largest biomedical databases, recruited over 500,000 participants from 22 recruitment centers in England, Scotland, and Wales between 2006 and 2010. All participants were of European ethnicity.^[20]^ ALM-related values were calculated as the sum of fat-free mass, measured using bioelectrical impedance analysis. GWAS summary data for low handgrip strength were derived from a meta-analysis of muscle weakness, including 256,523 participants.^[21]^ This meta-analysis synthesized data from 22 independent cohorts of European-descent individuals aged 60 years or older at assessment. Key cohorts included the UK Biobank, the US Health and Retirement Study, and the Framingham Heart Study. Maximum handgrip strength measurements were obtained for all the participants, serving as a critical indicator of muscle function. Muscle weakness was identified in 46,596 participants, defined according to EWGSOP criteria (male: <30 kg, female: <20 kg).

GWAS data for levels of 10 cathepsins were sourced from the INTERVAL study, which included 3301 European individuals.^[22]^ Blood donors aged 18 years or older were recruited at 25 centers of England’s National Health Service Blood and Transplant. Participants exhibited overall good health, attributed to the stringent blood donation eligibility criteria that excluded individuals with a history of significant medical conditions, such as myocardial infarction, stroke, malignancies, human immunodeficiency virus infection, and hepatitis B or C infection. Relative concentrations of plasma proteins were measured using the SOMAscan assay. This study was approved by the National Research Ethics Service (11/EE/0538), and informed consent was obtained from all participants. GWAS summary data for cathepsins are accessible via the IEU OpenGWAS Project. This study did not require ethical approval as it involved the use of de-identified data obtained from publicly accessible databases.

### 2.3. Instrumental variables

Instrumental variables (IVs) for the MR analyses were selected based on the following criteria to ensure instrument validity: First, IVs were chosen to ensure that the linkage disequilibrium measure among the instruments was <0.001 within a 10,000 kb window, minimizing potential correlations between IVs and enhancing instrument independence. Second, IVs were selected based on *P*-values below the genome-wide significance level identified in the corresponding study, which was set at 5 × 10^−6^. This threshold was established considering the limitations of the sample size to ensure that only statistically significant IVs were included in the analysis. Third, *F*-statistics were calculated to assess the strength of the selected IVs, with IVs exhibiting *F*-statistics < 10 considered weak instruments.

### 2.4. Statistical analysis

The inverse variance weighted (IVW) method was employed as the primary statistical approach to evaluate the causal relationship between cathepsins and sarcopenia-related traits. The Wald ratio method within the IVW framework was used to weigh the impact of each variant on the outcome, based on its effect on exposure. Individual MR estimates were combined to derive an overall summary value using a random-effects inverse variance meta-analysis. Complementary methods, including MR-Egger, simple mode, weighted median, and weighted mode, were utilized to validate the robustness of the MR results.

Sensitivity analyses were conducted to assess the validity of the causal relationships. The Cochrane *Q* test was used to estimate heterogeneity across IVs. A random-effects model of the IVW method was applied when significant heterogeneity was detected among the SNPs (*P*-value < .05). The MR-PRESSO method was used to identify outliers, and an MR pleiotropy test was conducted to detect horizontal pleiotropic effects.

## 3. Results

### 3.1. Influence of cathepsins on appendicular lean mass (ALM)

**Figure 2.**
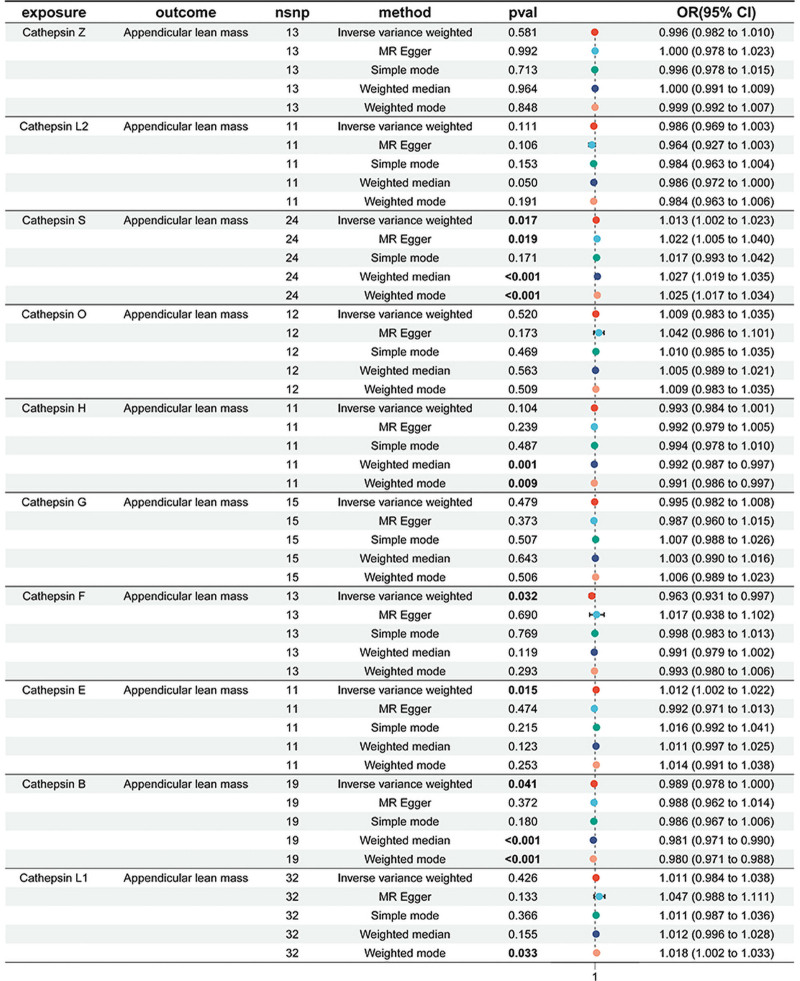
Mendelian randomization analyses of the causal effect of cathepsins on appendicular lean mass (ALM).

A two-sample MR Analysis was conducted to assess the effects of 10 cathepsins (cathepsins B, E, F, G, H, L2, O, S, L1, and Z) on ALM. Pleiotropy was indicated by the Cochrane *Q* test, prompting the use of a random-effects model in the IVW method (Table S1, Supplemental Digital Content, https://links.lww.com/MD/P77). Significant associations with ALM were identified for 4 cathepsins in the IVW analysis. Cathepsin S (odds ratio [OR] 1.013, 95% confidence interval [CI], 1.002–1.023; *P* = .017) and cathepsin E (OR 1.012, 95% CI, 1.002–1.022; *P* = .015) levels were positively associated with ALM. In contrast, cathepsin F (OR 0.963, 95% CI, 0.931–0.997; *P* = .032) and B (OR 0.989, CI 0.978–1.000; *P* = .041) were negatively associated with ALM (Fig. 2).

### 3.2. Influence of cathepsins on low handgrip strength

**Figure 3.**
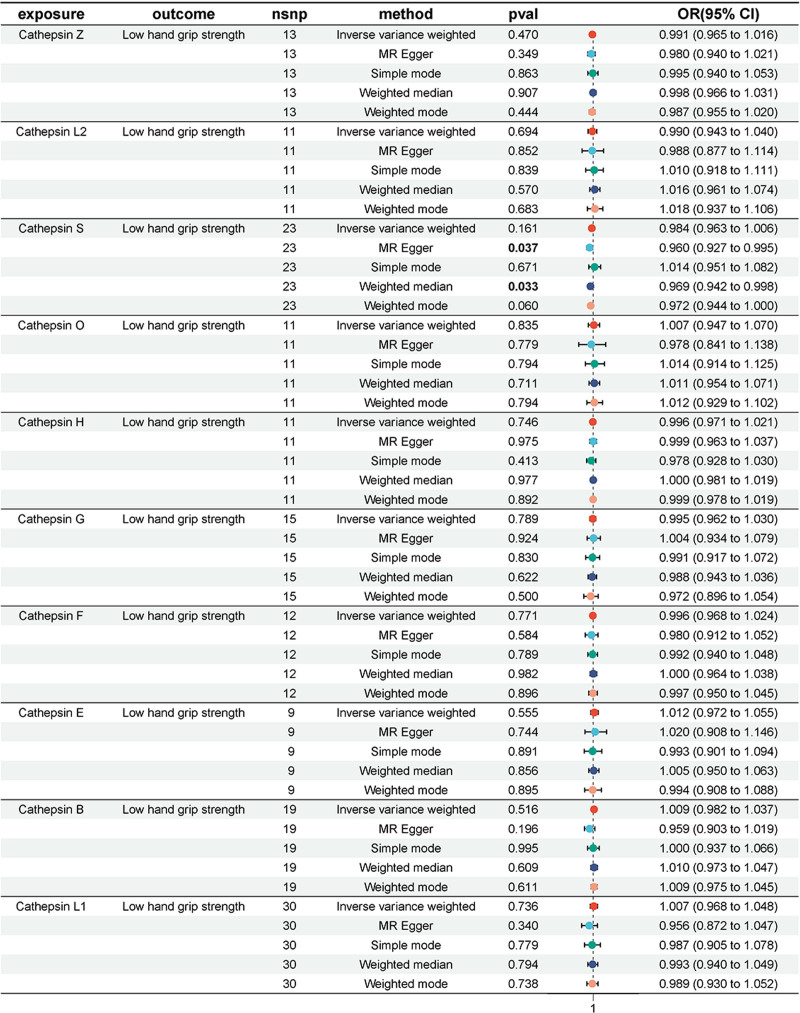
Mendelian randomization analyses of the causal effect of cathepsins on low hand grip strength.

A two-sample MR analysis was performed to evaluate the relationship between cathepsin and low hand grip strength. No significant associations were detected between cathepsin levels and low handgrip strength in the IVW analysis. However, MR-Egger (OR 0.960, 95% CI 0.927–0.995; *P* = .037) and weighted median analyses (OR 0.969, 95% CI 0.942–0.998; *P* = .033) found that cathepsin S was a risk factor for low handgrip strength (Fig. 3).

### 3.3. Sensitivity analyses

**Figure 4.**
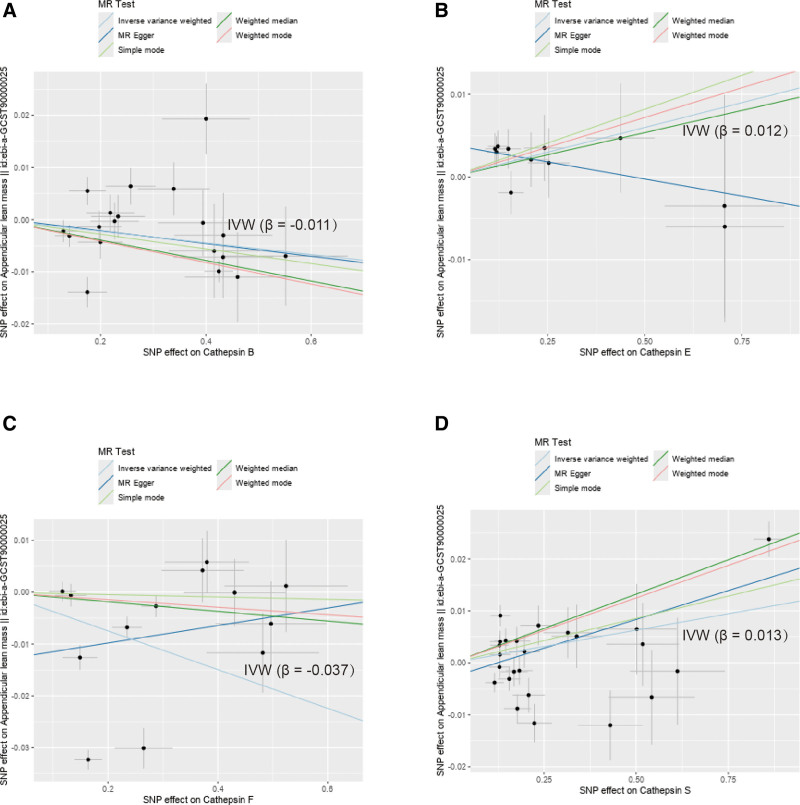
Scatter plots for the effects of SNPs (IVW). (A) SNPs of cathepsin B and ALM. Different Mendelian randomization (MR) methods (IVW, MR-Egger, weighted median, simple mode, and weighted mode) show consistent β values, all of which are negative, indicating that cathepsin B is negatively related to ALM. (B) SNPs of cathepsin E and ALM. IVW, weighted median, simple mode, and weighted mode show consistent β values, which are positive, indicating that cathepsin E is positively related to ALM. The MR-Egger test shows the opposite direction. (C) SNPs of cathepsin F and ALM. IVW, weighted median, simple mode, and weighted mode show consistent β values, which are negative, indicating that cathepsin F is negatively related to ALM. The MR-Egger test shows the opposite direction. (D) SNPs of cathepsin S and ALM. Different MR methods show consistent β values, all of which are positive, indicating that cathepsin S is positively related to ALM. ALM = appendicular lean mass, IVW = inverse variance weighted, SNP = single-nucleotide polymorphism.

**Figure 5.**
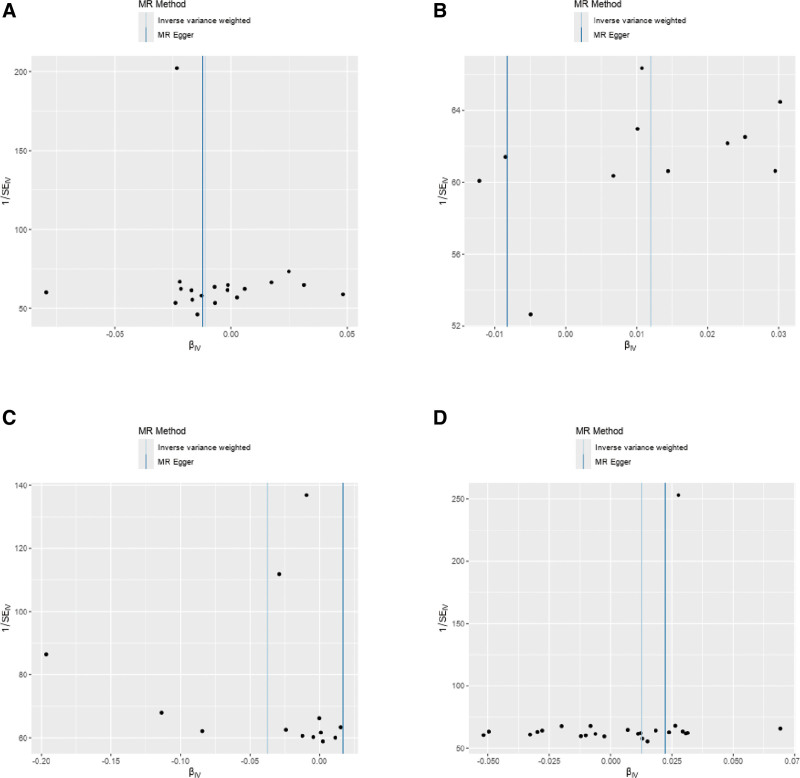
Funnel plot for SNPs. (A) SNPs of cathepsin B and ALM. (B) SNPs of cathepsin E and ALM. (C) SNPs of cathepsin F and ALM. (D) SNPs of cathepsin S and ALM. ALM = appendicular lean mass, SNP = single-nucleotide polymorphism.

**Figure 6.**
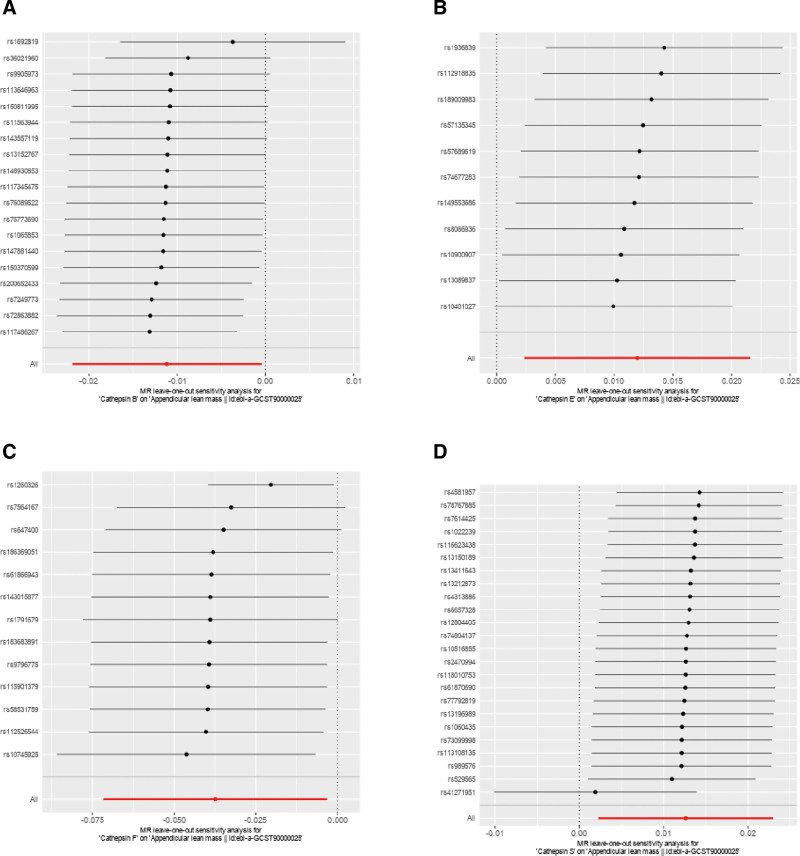
The “leave-one-out” analysis. (A) Cathepsin B on ALM. (B) Cathepsin E on ALM. (C) Cathepsin F on ALM. (D) Cathepsin S on ALM. ALM = appendicular lean mass.

No horizontal pleiotropy was detected in the MR-Egger intercept test for the IVs of the selected cathepsins, suggesting that the genetic variants used as IVs were not associated with confounding factors that could bias the results (Table S2, Supplemental Digital Content, https://links.lww.com/MD/P78). This finding supports the validity of the MR approach. Scatter and funnel plots were generated to further illustrate the robustness of the findings (Figs. 4 and 5). These visual representations underscore the stability of the data and clearly depict the relationship between cathepsins and sarcopenia-related traits. Scatter plots facilitated visual assessment of the associations, while funnel plots aided in identifying potential asymmetries indicative of publication bias or other issues (Figs. 4 and 5). Additionally, a leave-one-out analysis was conducted to evaluate the influence of individual SNPs on the MR results (Fig. 6). Each SNP was systematically removed to assess whether any single variant disproportionately affected the overall results. No single SNP associated with the cathepsin affected was found to alter the direction of the MR results, reinforcing the reliability of the conclusions.

## 4. Discussion

Sarcopenia, characterized by progressive loss of muscle mass, strength, and function, represents a significant public health concern, particularly among older adults. This study aimed to explore the causal relationships between cathepsins and sarcopenia-related traits through MR analysis. The primary findings revealed that specific cathepsins were significantly associated with ALM and, to a lesser extent, handgrip strength. These findings provide novel insights into the mechanisms by which cathepsins influence muscle health and highlight novel insights for therapeutic intervention.

Previous MR studies have established associations between cathepsins and cancer risk, though not specifically with muscle-related outcomes.^[23]^ For instance, elevated cathepsin H levels have been causally linked to an increased risk of lung cancer, particularly adenocarcinoma, in smokers, suggesting its potential as a biomarker for diagnosis and treatment.^[24]^ Our MR analysis revealed that cathepsins S and E were positively associated with ALM, whereas cathepsins F and B were negatively associated with ALM. The positive association of cathepsin S and E with ALM suggests a protective role in maintaining muscle mass. Cathepsin S, involved in extracellular matrix remodeling and immune response modulation, may contribute to muscle repair and regeneration.^[25,26]^ However, cathepsin S has also been implicated in chronic stress-induced skeletal muscle atrophy and dysfunction in mice, with its deficiency or inhibition shown to reduce muscle damage, metabolic imbalance, and apoptosis.^[10,27]^ Fukuda et al confirmed that cathepsin S activation contributed to elastin degradation and calcification in smooth muscle cells under high glucose conditions, highlighting its potential role in the progression of vascular calcification in diabetes.^[28]^ Accordingly, we speculate that cathepsin S acts as a context-dependent modulator in muscles, promoting regeneration under repair conditions but exacerbating loss during pathological states. Cathepsin E, an aspartic endopeptidase involved in the degradation of peptide bonds with a substrate specificity similar to that of cathepsin D,^[29]^ is expressed in various tissues, including immune cells, epithelial cells, and organs such as the spleen and lungs.^[23,30]^ Despite its involvement in immune responses and protein degradation, research on its role in muscle-related processes remains limited and requires further investigation. Combined with our MR Analysis results and the previous functions of cathepsin E, the role of cathepsin E in antigen processing and presentation may influence muscle health through immune system interactions.

Conversely, the negative associations of ALM and cathepsin F and B indicate potential roles in promoting muscle degradation. Cathepsin F, a human papain-like cysteine protease with unique substrate specificity, is highly expressed in tissues such as the heart, skeletal muscle, brain, and gonads. It is involved in protein degradation within acidic cellular compartments and is likely targeted to the lysosome through a signal peptide-independent pathway.^[31]^ Takaya et al have shown that cathepsin F promotes cellular senescence in skin cells, including fibroblasts and keratinocytes, and may serve as a therapeutic target for skin rejuvenation by eliminating aging cells.^[32]^ Although direct evidence on cathepsin F’s role in muscle tissues is limited, we can infer that cathepsin F might promote senescence and muscle degradation. For instance, cathepsin B has been implicated in proteolytic pathways that lead to muscle protein breakdown.^[33,34]^ Li et al showed that cathepsin B is a cysteine protease involved in protein degradation and has emerged as a potential diagnostic marker for skeletal muscle insulin resistance in type 2 diabetes, with its expression inversely correlated with insulin resistance markers in human skeletal muscle cells.^[35]^ These observations align with our findings, suggesting that cathepsin B is detrimental.

No significant associations were detected between cathepsin levels and handgrip strength in the primary IVW analysis. However, secondary analyses using MR-Egger and weighted median methods indicated that cathepsin S was a potential risk factor for reduced grip strength. This discrepancy underscores the complex relationship between cathepsin levels and muscle function. The involvement of cathepsin S in the inflammatory processes may contribute to muscle weakness by exacerbating muscle inflammation and damage.^[36]^ Supporting this, Wan et al demonstrated that cathepsin S contributed to chronic stress-induced skeletal muscle atrophy and dysfunction by disrupting protein metabolism and promoting apoptosis, suggesting cathepsin S inhibition as a potential therapeutic strategy for stress-related muscle diseases.^[10]^ Further investigation into the role of inflammation in sarcopenia and its impact on muscle strength is warranted.

One major strength of this study is its innovative application to explore the causal relationship between genetic variants of cathepsins and sarcopenia-related traits. By employing genetic variants as instrumental variables, MR overcomes the confounding and reverse causation issues that often limit observational studies. This approach allows for more robust causal inferences and provides stronger evidence than traditional epidemiological methods. The use of MR to investigate the specific effects of cathepsins on muscle health opens new avenues for understanding biological mechanisms underlying sarcopenia. The focused examination of individual cathepsins, specifically cathepsins S, E, F, and B, and their distinct roles in muscle metabolism provides a nuanced understanding of their contributions to muscle health. The positive associations of cathepsins S and E with ALM suggest protective roles in muscle preservation, whereas the negative associations of cathepsins F and B indicate potential contributions to muscle degradation. This understanding supports the development of targeted interventions to modulate cathepsin activity selectively for muscle preservation or mitigation of loss. The robustness of these findings was reinforced by rigorous sensitivity analyses, including tests for pleiotropy and heterogeneity. The absence of horizontal pleiotropy, confirmed by the MR-Egger intercept test, enhances the credibility of the results by ensuring that the instrumental variables are not influenced by external confounding factors. Additionally, the consistency of the results across various MR methods further enhanced the reliability of the study’s conclusions. Collectively, these methodological strengths not only bolster the validity of the study’s causal inferences but also provide a solid foundation for future research on sarcopenia and muscle-related diseases.

Despite these strengths, several limitations must be acknowledged. The genetic data used were predominantly derived from European populations, limiting the generalizability of the findings to other ethnic groups. Differences in genetic structure and gene–environment interactions across populations may affect the applicability of these results globally. Therefore, future research should replicate these findings in diverse cohorts to ensure broader relevance. Additionally, the study relied on summary-level genetic data for the MR analysis. While this data level is suitable for large-scale causal inference, it does not allow for the exploration of individual-level variability and micro-mechanisms, such as nonlinear gene-phenotype relationships or gene–environment interactions. Consequently, the accuracy of these estimates may be limited. Furthermore, although MR provides strong evidence of causality, it is fundamentally a statistical tool and cannot directly reveal the specific molecular mechanisms through which cathepsins influence muscle mass and function. Although the results are causally significant from a statistical perspective, translating these findings into actionable biological interventions requires functional studies, such as animal models and cellular-level investigations, to validate the underlying pathways and clarify the role of cathepsins in the regulation of muscle homeostasis. Integration of multi-omics data with experimental evidence is needed to bridge the gap between genetic associations and mechanistic understanding, facilitating the translation of sarcopenia interventions into clinical practice.

The use of samples from different databases introduces several potential limitations primarily related to population heterogeneity, measurement techniques, and study protocols. The use of bioelectrical impedance analysis for muscle mass measurement in the UK Biobank may introduce biases, as it is more sensitive to factors such as hydration status compared to the more accurate dual-energy X-ray absorptiometry. Additionally, the grip strength criterion for sarcopenia, based on EWGSOP standards, may not fully capture clinically relevant aspects of muscle weakness across diverse age groups and ethnicities. Furthermore, the interval study, despite providing valuable cathepsin data, had a relatively small sample size, limiting statistical power to detect small genetic effects. Future research should prioritize ethnic diversity and standardized, precise measurement techniques to enhance the reliability and applicability of findings.

## 5. Conclusion

This study provides robust evidence of the causal roles of specific cathepsins in sarcopenia-related traits. Cathepsins S, E, F, and B were identified as key regulators of muscle mass, offering novel opportunities for therapeutic intervention. These findings support the potential of targeting cathepsins to mitigate the adverse effects of sarcopenia, thereby enhancing the quality of life for affected individuals. Future research should focus on elucidating underlying biological mechanisms and validating these findings in diverse populations to facilitate the development of effective treatments for sarcopenia.

## Author contributions

**Conceptualization:** Yiwen Hou, Huaxin Deng.

**Data curation:** Yiwen Hou, Yan Liu.

**Investigation:** Wei Liu, Yan Liu.

**Methodology:** Wei Liu.

**Software:** Huaxin Deng.

**Supervision:** Yan Liu.

**Visualization:** Huaxin Deng.

**Writing – original draft:** Yiwen Hou, Wei Liu.

**Writing – review & editing:** Huaxin Deng, Yan Liu.

## Supplementary Material


